# Visualization of Atherosclerotic Plaques Paired with Joheksol 350 (Omnipaque)

**DOI:** 10.3390/biomedicines13020399

**Published:** 2025-02-07

**Authors:** Piotr Wańczura, Wiktoria Mytych, Dorota Bartusik-Aebisher, Dawid Leksa, Adrian Truszkiewicz, David Aebisher

**Affiliations:** 1 Department of Cardiology, Medical College of Sciences, The Rzeszów University, 35-310 Rzeszów, Poland; 2 English Division Science Club, Medical College, The Rzeszów University, 35-310 Rzeszów, Poland; wiktoriamytych@gmail.com; 3 Department of Biochemistry and General Chemistry, Medical College, The Rzeszów University, 35-310 Rzeszów, Poland; dbartusikaebisher@ur.edu.pl; 4 Rzeszów Center for Vascular and Endovascular Surgery, 35-310 Rzeszów, Poland; dleksa@gmail.com; 5 Department of Photomedicine and Physical Chemistry, Medical College, The Rzeszów University, 35-310 Rzeszów, Poland; atruszkiewicz@ur.edu.pl

**Keywords:** visualization, atherosclerotic plaques, MRI, iodine, atherosclerosis

## Abstract

**Background:** Cardiovascular disease is one of the leading causes of death around the globe. Atherosclerosis, a chronic inflammatory blood vessel disease that takes years to develop, is its primary cause. Instability and further plaque buildup are caused by chronic inflammation, which creates the conditions for possible rupture. The visualization of arterial lesions in situ can enhance understanding of atherosclerosis progression and potentially improve experimental therapies. Conventional histology methods for assessing atherosclerotic lesions are robust but are destructive and may prevent further tissue analysis. **Objectives:** The objective of the current study was to evaluate a novel, nondestructive method for the visualization and characterization of atherosclerotic lesions. **Methods and Results:** Thus, we tested the hypothesis that MRI paired with an iodine-based radiopaque stain would effectively characterize atherosclerotic plaques in a manner comparable to routine histology while maintaining sample integrity and providing whole-volume data.

## 1. Introduction

**Figure 1:**
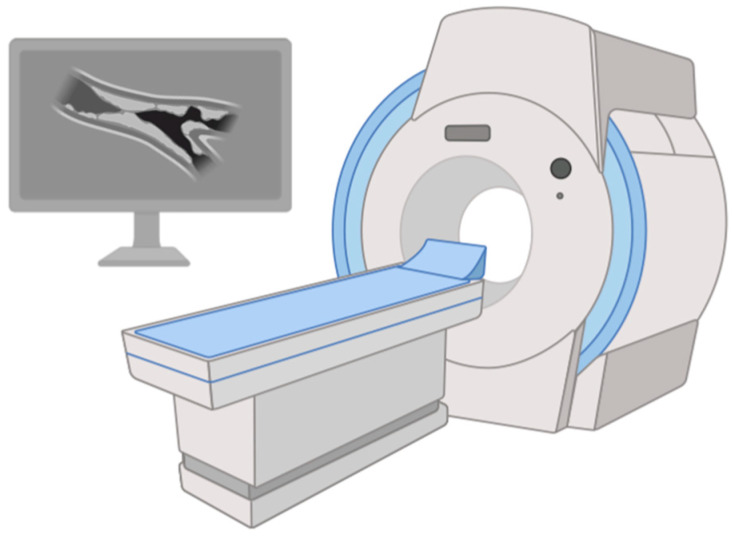
An example of MRI equipment for setup of atherosclerosis plaque imaging.

One of the leading causes of death around the globe is cardiovascular disease. Atherosclerosis, a chronic inflammatory blood artery disease that takes years to develop, is its primary cause [1,2]. It can be asymptomatic at first, which can make identification challenging [3]. Atherosclerosis causes the artery lumen to narrow as it advances, raising the risk of heart attacks, strokes, and other severe health issues. A stroke is a medical emergency in which the brain’s blood supply is cut off, leading to damage or death of brain cells. Fatty deposits in the arteries can cause atherosclerosis, which narrows or stops blood flow. This may cause the brain to receive less oxygen, which could lead to a stroke [4]. Furthermore, although it was often believed to be a disease that only affected the elderly, younger individuals are increasingly becoming affected as well [5]. Approximately 80% of deadly coronary thromboses are caused by ruptured atherosclerotic plaques. Men are more frequently impacted than women. It has been proposed that the disease develops because of both environmental and genetic causes [6,7]. Atherosclerosis is characterized by the development of so-called atherosclerotic plaques in the artery walls. Over time, this process leads to structural alterations that obstruct blood flow [8]. Hypertension, diabetes, hypercholesterolemia, obesity, and smoking are important contributors to the development of atherosclerotic plaques. Environmental pollution, sleep problems, and physical inactivity also have an impact on the development of atherosclerotic lesions [9,10,11]. In its 2022 statement, the European Atherosclerosis Society noted that elevated levels of lipoprotein(a) are particularly significant in the development of atherosclerosis and have been found to be a contributing factor to the disease [12]. Extensive studies have been conducted for many years to determine the precise mechanism of atherosclerotic lesions or the pathophysiology of atherosclerosis [13]. The alterations that lead to the development of plaques start with the inflammatory process. Fatty and fibrous debris is deposited in the artery walls because of an interaction between risk factors and arterial wall cells [14,15]. Lesions develop and vessel walls harden over time, which can totally stop blood flow [16]. Both invasive and non-invasive techniques are used to diagnose atherosclerosis to gauge the disease’s severity and potential for consequences. Computed tomography (CT), nuclear scanning, magnetic resonance imaging (MRI), blood biomarkers, and stress tests to measure coronary blood flow are examples of non-invasive diagnostics [17,18]. For several reasons, investigating T_1_ and T_2_ relaxation times in atherosclerosis is novel and highlights their potential as predictive and diagnostic instruments in cardiovascular disease. Researchers can now obtain far more specific information regarding tissue composition thanks to recent developments in MRI techniques (Figure 1), sophisticated pulse sequences, and enhanced spatial and temporal resolution [19]. These developments make it possible to measure the T_1_ and T_2_ relaxation durations in plaques and the arterial wall with greater sensitivity and accuracy. To evaluate the microstructural heterogeneity of atherosclerotic plaques and correlate changes in relaxation time with underlying tissue properties like inflammation, calcification, or lipid content, high-resolution imaging is very important [20,21]. Current research is starting to concentrate on the relationship between plaque components and T_1_ and T_2_ relaxation times. Researchers are starting to create more accurate non-invasive methods to evaluate plaque vulnerability by linking T_1_ and T_2_ times to certain plaque components. This could help detect plaques at risk of rupture before clinical events take place. The non-invasive visualization of atherosclerotic plaques and their components has become a cornerstone of modern cardiovascular medicine. However, imaging soft tissues, especially the vascular walls and atherosclerotic lesions, can be challenging due to the inherent low contrast between soft tissues. In this context, optical coherence tomography (OCT) offers a promising alternative to traditional imaging modalities, providing high-resolution, cross-sectional images of arterial structures without the need for extensive contrast agents or staining procedures [22,23,24,25]. OCT operates based on light scattering rather than radiation, enabling it to provide detailed tissue analysis at the microscopic level and making it an invaluable tool in the assessment of atherosclerosis. In the context of atherosclerosis, OCT has proven particularly useful in imaging the arterial wall and the components of atherosclerotic plaques, such as fibrous tissue, lipid cores, calcifications, and the fibrous cap [26,27,28]. Unlike CT, OCT offers much higher resolution, typically in the range of 10–20 microns, allowing for precise imaging of plaque morphology, a key factor in determining plaque stability and the risk of rupture [29,30]. However, OCT also faces some limitations, particularly in its ability to visualize deep tissue structures and certain plaque components. The primary challenge with OCT lies in the low intrinsic contrast of soft tissues, like the issues encountered in CT imaging [31,32]. While OCT is excellent for visualizing the microstructure of the vessel wall, it can struggle to distinguish different soft tissue components within the plaque. To overcome this limitation, OCT may utilize contrast agents that enhance the visibility of specific tissue structures, providing additional information about plaque composition and vulnerability [33,34]. Optical coherence tomography contrasts differ from those used in CT or MRI, as they often involve the use of exogenous agents designed to enhance tissue contrast through scattering rather than relying on density differences as in radiographic imaging. Several types of contrast agents have been developed specifically for OCT [35,36]. Fluorescent and micron-sized particles are often designed to target specific plaque components, such as macrophages, to identify regions of inflammation. For example, fluorescent nanoparticles that bind to inflammatory markers in the plaque can provide detailed insights into the cellular composition of the lesion [37,38]. Intravenous dye-based agents, such as indocyanine green (ICG), are sometimes used in OCT to enhance the imaging of endothelial cells and blood vessels. Although these dyes are more commonly used in ophthalmic OCT, their potential in cardiovascular imaging is still under exploration [39,40]. The use of molecularly targeted contrast agents that specifically bind to atherosclerotic plaques is an emerging field. These agents could enhance the detection of vulnerable plaques by targeting macrophages or oxidized LDL, which are markers associated with inflammation and plaque instability [41]. The advantage of using contrast agents in OCT is their ability to enhance the contrast of specific tissue types, such as the fibrous cap or lipid-rich necrotic core, improving the accuracy of plaque characterization. This approach helps distinguish between stable and unstable plaques, which is critical for risk stratification in patients with coronary artery disease [42,43]. OCT offers detailed imaging of atherosclerotic plaques, revealing key structural features that contribute to plaque stability. The ability to assess these features in real time is crucial for identifying plaques that may be at risk of rupture and subsequent thrombotic events [44]. The fibrous cap is the outer layer of an atherosclerotic plaque and plays a crucial role in determining plaque stability. A thin or ruptured fibrous cap is a hallmark of vulnerable plaques, which are more likely to rupture and cause an acute coronary event [45,46]. OCT allows for the precise measurement of fibrous cap thickness and the assessment of its integrity, making it one of the most important features in plaque vulnerability assessment [47]. The lipid-rich necrotic core is another important component of atherosclerotic plaques, and its size and composition can influence plaque stability. On OCT, the lipid core typically appears as a hypointense region (dark area), while the fibrous cap appears hyperintense (bright). Changes in the size or composition of the lipid core can be indicative of plaque progression or destabilization [48,49,50]. OCT is also capable of visualizing calcifications, which are often present in advanced stages of atherosclerosis. These calcified areas appear as bright spots on OCT images due to their high scattering properties. Calcifications are important for assessing plaque stability, as they may contribute to plaque rigidity and reduce the likelihood of rupture [51].

The current study set out to assess a new, nondestructive technique for atherosclerotic lesion MR imaging with Omnipaque 350; we also present the optical coherence tomography results.

## 2. Materials and Methods

### 2.1. Materials

During experiments presented in this paper, all samples were placed in Eppendorf^®^ 0030 000.919 tubes (Sigma Aldrich, Warsaw, Poland). We used Joheksol 350 (Omnipaque 350 mg I/mL) produced by General Electric HealthCare (Munchen, Germany). For the preparation of a suitable concentration of Omnipaque solution, we used the AquaB Duo reverse osmosis system manufactured by Fresenius Medical Care in Singapore. Shortly after samples were collected, MRI acquisition experiments were performed with a 1.5 Tesla OPTIMA 360MR manufactured by General Electric HealthCare Munchen, manufactured in Munchen, Germany. In this research, we used a balance from Ohaus PX224 (Geifensee, Switzerland). The room temperature during all performed experiments was 18 °C.

### 2.2. Methods

#### 2.2.1. Plaque Tissue Sample Preparation for MRI Measurement

Tissue samples of arteries with atherosclerotic lesions were collected by endarterectomies from 7 patients (5 male and 2 female). Patients qualified for endarterectomy were in the age range of 69–81 years and had experienced an episode of ischemic stroke. The inclusion criteria for the study plaque analysis were a history of ischemic stroke due to critical carotid artery stenosis and qualification for surgical treatment by endarterectomy. The exclusion criteria were the following: other than normal sinus rhythm, for example, history of atrial fibrillation/flutter as a possible reason for ischemic stroke, and qualification for other than a surgical way of carotid artery stenosis treatment. The significance of carotid artery stenosis must have been confirmed by angio CT (computed tomography). Samples were excised from the carotid artery, which had previously been image-diagnosed and finally qualified for removal. All bioethical consents presented in this research were performed according to Bioethical Certificate no 43/2024/B issued by the Bioethics Committee of the Rzeszow Regional Medical Chamber.

Fourteen samples (two from one patient; the number of patients was seven) were evaluated. Only plaques from patients undergoing surgical endarterectomies due to ischemic stroke were qualified for further assessment (Table 1). The history of supraventricular arrythmias as atrial fibrillations and low ejection fraction due to myocardial infarction were excluded from the study pool in order to concentrate only on ischemic stroke due to significant/critical carotid artery stenosis. The history of ischemic stroke must have been confirmed based on available medical records, including CT scan assessments. The diagnostic tool was the Philips AFFINITI 50 ultrasound machine with a C6-2, S4-2, L12-4 transducer. The patients were operated on at the Department of Vascular Surgery of the Polish-American Heart Clinics PAKS IX in Rzeszów. Each patient was informed in detail about ischemic stroke resulting from carotid artery stenosis. All patients were informed about both the benefits and complications associated with the procedure, to which they gave written consent. Patients included in the study were all Caucasians. All patients were diagnosed with hemodynamically significant stenosis of the right or left carotid artery. Before the procedure, patients received acetylsalicylic acid and statins according to the recommendations for endarterectomy procedures.

**Figure 2:**
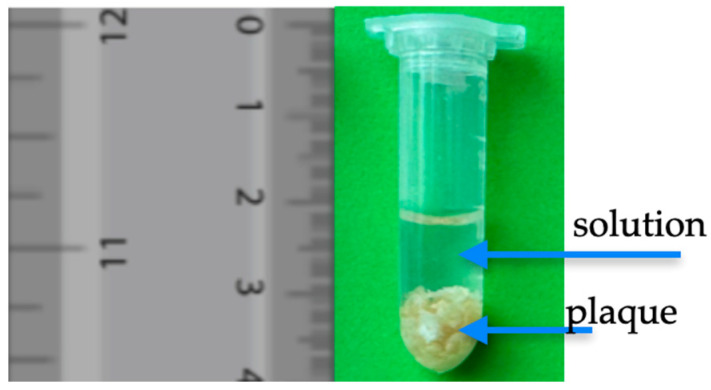
An example of the setup of an atherosclerosis plaque in an Eppendorf tube in a 1.6 mL solution.

The collected samples were placed in test tubes with physiological saline, which were then subjected to in vitro testing. The samples were measured with and without the Omnipaque 350 contrast. Omnipaque 350 was administrated according to the procedure below. The collected atherosclerotic lesions had weights of 0.2354 g, 0.4393 g, 0.2747 g, 0.412 g, 0.3368 g, 0.1991 g, 0.5710 g, 0.442 g, 0.312 g, 0.389 g, 0.258 g, 0.483 g, 0.294 g, and 0.304 g. The samples (Figure 2) were measured by Ohaus PX224 (Geifensee, Switzerland).

**Figure 3:**
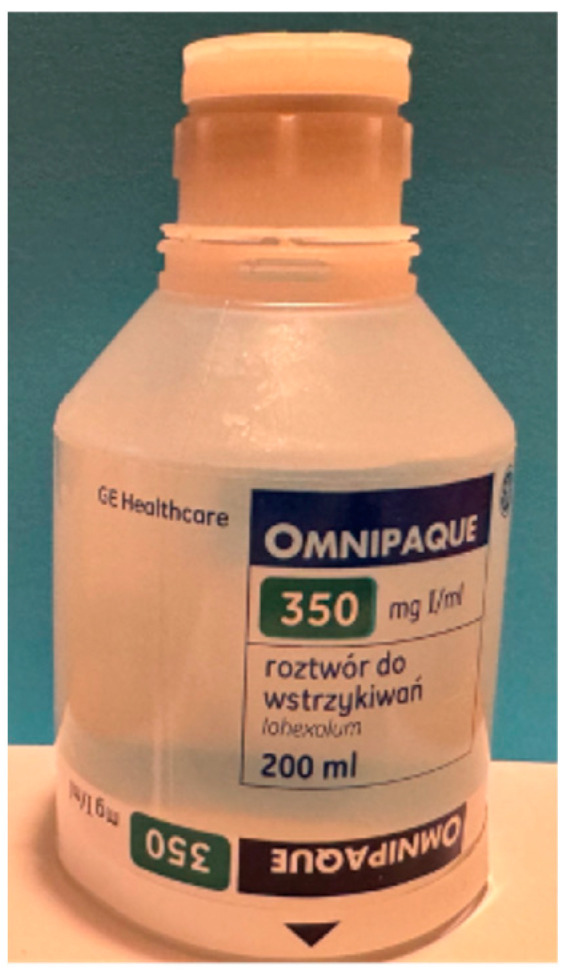
Joheksol 350 (Omnipaque 350 mg I/mL) manufactured by General Electric HealthCare (Munchen, Germany) [52].

In brief, a concentration of 4 µL Omnipaque 350 mg I/mL manufactured by General Electric HealthCare (Munchen, Germany) (Figure 3) was applied to the atherosclerotic plaques. From the predecessor’s protocol, the preferred in vivo dose in humans for the external carotid artery is 6–9 mL, and for the internal carotid artery is 8–10 mL. Omnipaque 350 contrast was added to the test samples to study the possible absorption of Omnipaque 350 and its possible visible effect on T_1_ and T_2_ relaxation times. A 24 h incubation time was used to allow Omnipaque 350 contrast to penetrate and accumulate. Extended incubation allows iodine molecules to perfuse the atherosclerotic plaque and enhance better signal intensity on MRI images, allowing a more detailed visualization of the plaque. The concentration used in the study was calculated from a clinical dose provided by the contrast manufacturer, as mentioned above. Samples were immersed in 1.6 mL of distilled water + 4 µL of Omnipaque 350. In our laboratory, water for the preparation of stock solutions from commercially obtained Omnipaque 350 was purified using an AquaB Duo reverse osmosis system from Fresenius Medical Care, Singapore.

#### 2.2.2. MRI Examination

**Figure 4:**
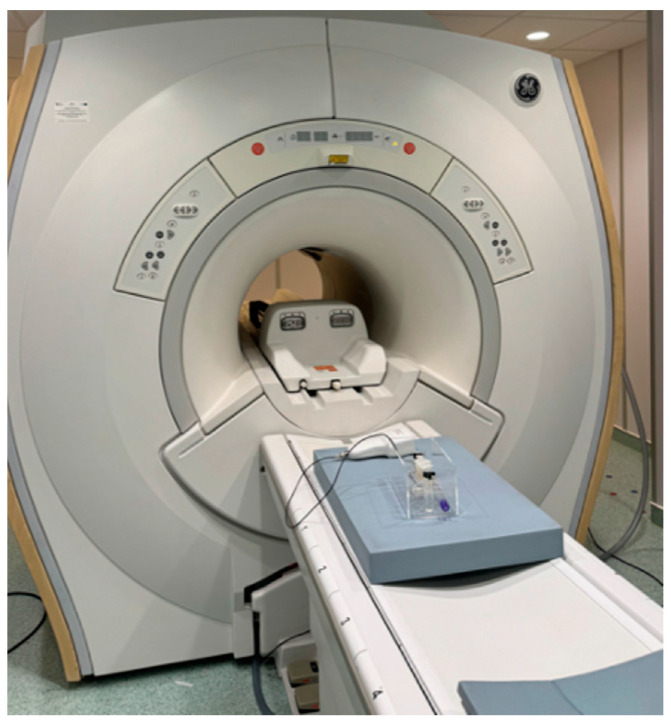
MR imaging.

The samples were left in Omnipaque 350 mg I/mL solution for 24 h at room temperature. After 24 h, the samples were subjected to magnetic resonance imaging (MRI). The study was designed to determine longitudinal (T_1_) and transverse (T_2_) relaxation times. It was performed using a clinical magnetic resonance system—OPTIMA 360M R prod. GEHC (Milwaukee, WI, USA) (Figure 4). The system used for the study had a 1.5T superconducting magnet. The device used software version SV23. For the test, a coil was used that allowed for flexibility, allowing it to better fit the test items, which are the test tubes used. This approach allowed the imaging capabilities of the system to be maximized. The study was performed using standard echo sequences. As a result of image acquisition, material was obtained for further processing, because of which the numerical values of T_1_ and T_2_ times and the distribution of these times in the test space were obtained. In addition, the distribution of the R2 coefficient was obtained, which illustrates the accuracy of fitting the approximating exponential function to the numerical data.

By progressively increasing the duration, the repetition time-TR was evaluated for the T_1_ measurements in the 50–15,000 ms range. In all, twelve trials were conducted. From the following range of values—all measured in milliseconds—the TR time value was progressively chosen: 50; 100; 200; 300; 500; 1000; 1500; 2000; 3000; 5000; 10,000; and 15,000. Every stage of the MRI examination was conducted using the same technical settings. The field of view (FOV) was 6 cm × 6 cm, the scan matrix was 256 × 256 pixels, the section thickness was 1 mm, the spacing was 0.5 mm, and NEX = 2. Three milliseconds was the constant echo-TE time. The MR images of the tested samples and their parameters were obtained by manipulating the TR-time settings to obtain the fast spin-echo (FSE) sequence. SNR is increased and examination time is decreased with fast spin-echo (FSE) imaging. The following steps made up the test protocol for figuring out the T_1_ relaxation time: system calibration; recognition sequence, which involves finding an object in three planes (frontal, sagittal, transverse, and 3-plane); and coronal sequence (T_1_) of the fast spin-echo—FSE (with varying repetition time-TR) values. Various spin-echo (TE) periods were employed to measure the relaxation time T_2_. With the same scanning parameters, 12 steps with varying TE times (from 1 ms to 250 ms) were employed. The echo time ranged from 11.8 to 300 ms (11.8, 20, 42, 68, 85, 102, 130, 160, 200, 230, and 250 ms), but the repetition time remained constant at 10,000 ms. The repetition time in this setup remained constant at 15 × 103 ms. The T_2_ FSE frontal sequence, not the T_1_ FSE, was the third stage in the measurement process, whereas the subsequent phases were the same as for the T_1_ relaxation time (system calibration and recognition sequence). Both pre-Omnipaque 350 and post-Omnipaque 350 tissues underwent MR scanning. A signal intensity versus time curve fitting model might then be used to calculate the T_1_ values pixel-by-pixel. A signal intensity versus echo time curve fitting mode might then be used to calculate the T_2_ values pixel-by-pixel.

#### 2.2.3. Optical Coherence Tomography

Intravascular optical coherence tomography allows for the accurate analysis of the composition of atherosclerotic plaque in individual sections. The study involves spiral scanning of a vessel with a beam of homogeneous light with a wavelength corresponding to near-infrared and analyzing the spectrum of the reflected light. Since the frequency of electromagnetic waves is many times higher than that of ultrasound, the resolution of images generated by this technique is also much higher. The smallest element identified as a single point is only 10 μm in size, which is sufficient for the precise imaging of atherosclerotic plaques, including the measurement of the thickness of the surface fibrous layer. OCT allows for a vivid view of the atherosclerotic plaque and the identification of unstable lesions.

#### 2.2.4. Statistical Analysis

For each sample of atherosclerotic plaque used in this study, a two-way ANOVA test was performed to test for the simultaneous effects of time after treatment on the outcome variables. Statistical significance was defined as *p* < 0.05. All MRI data are reported as mean ± SD. The data were analyzed using Statistica 13.1 software (StatSoft Polska Sp.z o.o., Krakow, Poland).

## 3. Results

Table 1 presents the characteristics of the study group. The number of cases (n) and total number of patients (N) are provided for each category.

### 3.1. MRI Results

**Figure 5:**
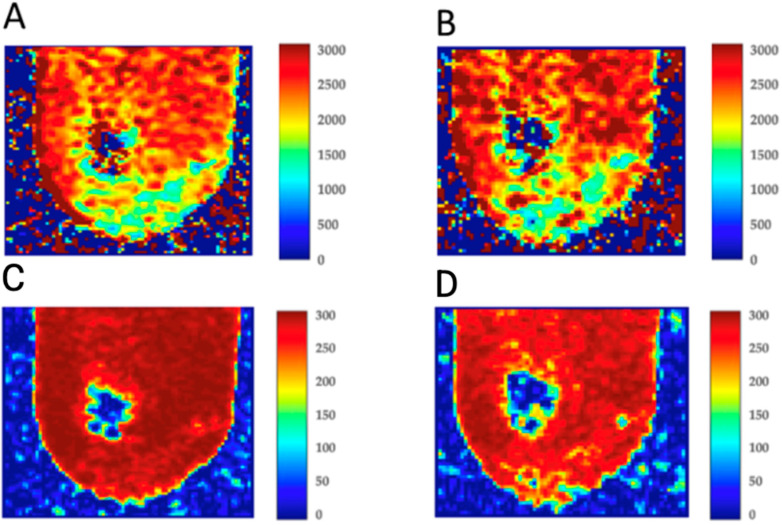
T_1_ and T_2_ distribution in human plaques. (**A**) T_1_ before Omnipaque 350 administration; (**B**) T_1_ 48 h after Omnipaque 350 administration; (**C**) T_2_ before Omnipaque 350 administration; (**D**) T_2_ 48 h after Omnipaque 350 administration.

In this study, we analyzed the effect of Omnipaque 350 administration on the T_1_ and T_2_ relaxation times of human atherosclerotic plaques, with a particular focus on the alterations in the spatial distribution and concentration of the contrast agent within the plaques. The average T_1_ and T_2_ relaxation times for each experimental step are summarized in Table 2, which serves as a basis for understanding the dynamics of water content and Omnipaque 350 distribution within the plaques. Figure 5 illustrates typical T_1_ and T_2_ relaxation time distributions in human plaques, obtained before and 48 h after the administration of Omnipaque 350, respectively.

The qualitative differences in T_1_ and T_2_ values highlight the impact of Omnipaque 350 on the biochemical and biophysical properties of atherosclerotic plaques. Specifically, the MRI data indicate that the distribution of the contrast agent in plaques alters both T_1_ and T_2_ relaxation times, reflecting significant changes in the tissue’s water content and the presence of the contrast agent. Before contrast administration, the plaques exhibited higher T_1_ and T_2_ values, indicative of higher water content and less restricted molecular motion. This is consistent with the characteristics of atherosclerotic plaques, which typically contain substantial amounts of water in their extracellular matrix. Following the administration of Omnipaque 350, significant changes in both T_1_ and T_2_ relaxation times were observed, which can be attributed to the higher concentration of Omnipaque 350 within the plaques. The contrast agent’s presence is known to affect proton relaxation, primarily by altering the local magnetic field interactions in water molecules, leading to faster relaxation times in the presence of Omnipaque. As expected, following the equilibration period of 48 h, the Omnipaque 350 concentration within the plaques was notably higher, as evidenced by the marked reduction in T_1_ and T_2_ relaxation times when compared to pre-contrast values. The spatial distribution of Omnipaque 350 within the plaques was further assessed, revealing a distinct pattern. In regions with higher Omnipaque 350 accumulation, there was a corresponding decrease in T_1_ and T_2_ relaxation times, suggesting a localized reduction in water content and an increase in the concentration of the contrast agent. This finding is consistent with the known behavior of contrast agents in biological tissues, where their presence often leads to a reduction in water proton relaxation times due to their paramagnetic properties. Interestingly, the plaques demonstrated a more heterogeneous distribution of Omnipaque 350 after 48 h, with some areas showing significantly lower relaxation times than others. This heterogeneity could be indicative of varying degrees of plaque composition, such as lipid content, fibrous tissue, and necrotic core, all of which can influence the way contrast agents distribute within the plaque. The altered relaxation times are reflective of changes in the molecular environment within the plaques, where areas with higher concentrations of Omnipaque 350 tend to show faster relaxation times, and areas with higher water content, which retain less contrast agent, exhibit slower relaxation times. Both T_1_ and T_2_ relaxation times provide valuable insights into the tissue composition, especially regarding water content (Table 3). Longer T_1_ and T_2_ times are generally associated with higher water content and less restricted molecular motion, whereas shorter times indicate a lower water content and more restricted motion, often due to the presence of macromolecules or contrast agents like Omnipaque 350. The significantly longer T_1_ and T_2_ relaxation times observed in the control plaques suggest that they retained a higher water content compared to the treated plaques, which had absorbed the contrast agent and exhibited a reduction in water content as a result. This decrease in relaxation times following Omnipaque 350 administration is likely due to the contrast agent’s effect on the plaques’ structural and chemical composition. The presence of Omnipaque 350 leads to a reduction in the molecular freedom of water molecules within the plaque, which is reflected in the shortened T_1_ and T_2_ values. These changes are important because they offer insight into the potential of contrast agents like Omnipaque 350 to influence plaque characteristics, which may have implications for both diagnostic and therapeutic strategies in cardiovascular disease. The results of this study underscore the sensitivity of T_1_ and T_2_ relaxation times to changes in plaque composition and the distribution of contrast agents. The marked reductions in T_1_ and T_2_ times following Omnipaque 350 administration provide compelling evidence of its uptake by atherosclerotic plaques, resulting in a decrease in water content and an alteration in tissue characteristics. These findings have significant implications for the non-invasive imaging of atherosclerotic plaques, as changes in MRI-derived parameters such as T_1_ and T_2_ relaxation times can serve as biomarkers for plaque composition and the effectiveness of contrast agents in enhancing plaque visualization. Future studies may further investigate the relationship between these MRI parameters and the histopathological features of plaques, as well as explore the potential of Omnipaque 350 and other contrast agents in monitoring plaque stability and the risk of rupture.

In this study, we observed significant differences in the median T_1_ and T_2_ relaxation times of plaques before and after the administration of Omnipaque 350, a contrast agent. Our findings indicate that Omnipaque 350 influences the relaxation times in a manner that is dependent on its penetration depth in the plaque. In human plaques, both T_1_ and T_2_ relaxation times typically decreased as the contrast agent infiltrated deeper into the plaque. These changes reflect underlying alterations in the plaque’s biochemical composition. The reduction in relaxation times suggests that Omnipaque 350 may be altering the water content, the tissue microstructure, or the molecular environment within the plaque, which, in turn, could modify the signal intensity observed on MRI scans. A key observation from this study was the discrepancy between T_1_ and T_2_ relaxation times measured at two different time points: 1 h and 48 h post-Omnipaque 350 administration. This temporal variation in the relaxation times may reflect ongoing physiological processes, such as the redistribution or washout of the contrast agent, or more complex tissue responses to contrast medium infiltration. These differences are not simply technical or artifact-driven but are statistically significant, indicating that they likely represent true biochemical differences within the plaque tissue. The contrasting patterns observed between T_1_ and T_2_ values could also suggest differential effects of Omnipaque 350 on the plaque’s lipid-rich core versus the fibrous cap, highlighting the heterogeneous nature of plaque structure and its response to contrast enhancement. Furthermore, the changes in T_1_ and T_2_ relaxation times that we observed are not merely an artifact of contrast administration but are indicative of alterations in the fundamental tissue properties of human plaques. This suggests that MRI, when combined with contrast agents like Omnipaque 350, can provide a more detailed and nuanced characterization of plaque composition, structure, and stability. Such information is critical for assessing the risk of plaque rupture, which is a major cause of acute cardiovascular events. The ability to non-invasively monitor plaque progression and composition using MRI could thus have significant implications for clinical practice, aiding in the risk stratification of patients with atherosclerosis and potentially guiding therapeutic decisions. Our results encourage further investigation into the use of MRI, with particular attention to contrast agents like Omnipaque 350, in the assessment of human plaques. Future studies should explore the relationship between T_1_ and T_2_ relaxation times and the specific histopathological features of plaques to better understand the underlying mechanisms driving plaque vulnerability. Additionally, a more comprehensive study design that incorporates a larger cohort of patients and a longer follow-up period would help validate the clinical utility of MRI as a tool for monitoring plaque stability and progression in atherosclerotic disease.

### 3.2. Imaging of Coronary Artery In Vivo

**Figure 6:**
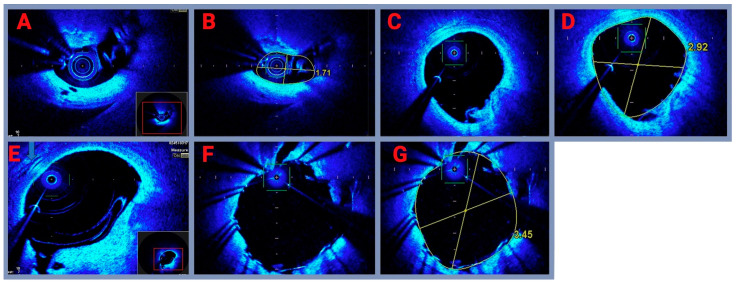
OCT assessment of coronary artery plaque in vivo before and after intravascular lithotripsy from one of the patients (IVL). (**A**,**B**) Critical LM stenosis with transactional area measurement; (**C**,**D**) IVL results with transactional area measurement; (**E**) calcium tissue defragmented by IVL; (**F**,**G**) final result after stent implantation with transactional area measurement.

To investigate the changes in the left main coronary artery (LMCA) during coronary angioplasty, optical coherence tomography (OCT) technology was used to capture detailed imaging at various stages of treatment. The imaging process is illustrated in Figure 6. Left main before treatment (Figure 6A): this image shows the left main coronary artery before any interventions, with no measurements provided. The OCT probe is centered in the middle, surrounded by the initial segment of the left coronary artery. In the upper section of the image, a transverse view is presented, while the lower section shows a longitudinal view of the artery. Left main with measurements (Figure 6B): a marked stenosis is present in the left main coronary artery, with a yellow outline highlighting the artery’s borders. The minimal lumen area (MLA) is measured at 1.4–3.5 mm², indicating the presence of significant narrowing. Left main after intracoronary lithotripsy without measurements (Figure 6C): the image immediately after intracoronary lithotripsy therapy, showing a noticeable increase in the minimum lumen area to 5.2 mm². This demonstrates the successful expansion of the artery following the procedure. Left main after intracoronary lithotripsy with measurements (Figure 6D): after lithotripsy, the image shows the calcium deposits within the artery. The deposits are clearly fragmented, indicating successful microfracturing by the lithotripsy procedure. Left main after drug-eluting stent (DES) placement (Figure 6E): a magnified view of the left main coronary artery shows the stent placed in the vessel with the visible deployment and expansion of the stent struts, which are outlined by a yellow circle. Left main after stenting with measurements (Figure 6F): following stent implantation, the OCT image shows well-expanded stent struts and a properly dilated vessel. This section illustrates the final angiographic result post-stenting. OCT imaging effectively captured the stages of coronary angioplasty, from pre-treatment conditions to post-stent implantation. The imaging clearly highlights the treatment process, including the lithotripsy procedure’s role in fragmenting calcium deposits and the success of the stent in restoring the vessel lumen and achieving optimal angiographic results.

## 4. Discussion

MRI is the most used modality for the non-invasive detection of changes in solid and liquid biological samples. In this study, specific MRI parameters, such as T_1_ and T_2_ measurements, have been correlated with the tissue changes associated with tissue contrast administration. The MRI-measured parameters T_1_ and T_2_ have been shown to respond to Omnipaque 350 implementation and changes in hydration, respectively.

The identification of an atherosclerotic plaque and its structure is crucial for successful treatment. Raising different parameters of plaque detection may play an important role in choosing the most effective diagnostic and therapeutic method. In the presented study, the average of the T_1_ and T_2_ relaxation times on the carotid atherosclerotic plaque are analyzed in correlation with the usage of one of the most popular invasive radiology iodine contrast solutions (Omnipaque 350). The implementation of the aforementioned contrast resulted in a decrease in the T_1_ and T_2_ relaxation times in the observed periods 1 h and 48 h after Omnipaque 350 implementation (T_1_ slope from 1231 ms to 460 ms, T_2_ slope from 63 ms to 33 ms). The obtained changes suggest that MRI assessment after the usage of iodine contrast may deliver additional information about atherosclerotic plaque distribution and differentiation toward more effective, conservative, pharmacological treatment of soft plaques versus more calcified plaques resistant to pharmacology—mostly sclerotic or calcified plaques. The structure of atherosclerosis could be visualized in the invasive procedure as well. OCT is a diagnostic procedure with the highest spectral resolution; however, it requires artery catheterization with intraarterial iodine contrast delivery. The plaque burden, deposits of calcium before and after debulking procedures such as rotational or orbital atherectomy, plaque dissections, and finally, the apposition of stent struts after artery stenting may deliver additional information for minimalizing the risk of early restenosis or intrastent thrombosis. In the presented study, the typical usages of OCT in intravascular plaque treatment are presented with excellent results for final stent apposition. The results of our experimental work underline the further investigations that are still needed in atherosclerotic plaque structure to obtain the most effective multidisciplinary approach in multilevel atherosclerosis, including invasive and aggressive pharmacological modifications.

The main treatment for atherosclerosis combines lifestyle changes, medications, and potential procedures or surgeries to manage plaque buildup in arteries, reduce cardiovascular risks, and improve blood flow. Lifestyle modifications are essential for slowing atherosclerosis progression. A diet low in saturated fats, cholesterol, and refined sugars; regular exercise; weight management; and smoking cessation help reduce blood pressure, lower LDL (bad) cholesterol, and improve overall cardiovascular health [53,54]. Medications are often prescribed to manage risk factors, such as statins to lower cholesterol, antihypertensive drugs to control blood pressure, and antiplatelet agents like aspirin to reduce blood clot risk. In patients with diabetes, drugs to regulate blood sugar are also critical [55,56]. For severe atherosclerosis where blood flow is significantly obstructed, procedures like angioplasty or surgical interventions may be necessary. Angioplasty involves inserting a balloon or stent to widen narrowed arteries [57,58]. Coronary artery bypass grafting creates new pathways around blocked arteries to restore blood flow [59]. Another procedure, endarterectomy, is used to directly remove atherosclerotic plaques from larger arteries, such as the carotid artery, to prevent strokes. In an endarterectomy, the surgeon carefully excises the plaque from within the artery, reducing the risk of future blockages [60,61]. These treatments, combined, address symptoms and complications of atherosclerosis, prevent further plaque buildup, and improve patient outcomes, particularly when paired with ongoing lifestyle and medical management. T_1_ time and T_2_ time imaging sequences are the most useful MRI techniques for determining the properties of atherosclerotic plaques. Predicting the likelihood of plaque rupture and subsequent thrombotic events requires complementing information regarding the tissue composition and stability of the plaques, which these sequences offer. The way MRI functions is by identifying the body’s protons’ magnetic characteristics [62,63]. These protons’ behavior in a magnetic field, which is utilized to create images, is influenced by the tissue’s characteristics, such as its fat and water content. By varying the timing of radiofrequency pulses, the MRI signal can be altered, producing several image types based on the pulses’ timing. The various components that make up atherosclerotic plaques are heterogeneous and can be seen differently with T_1_ and T_2_ MRI [64]. Understanding plaque composition and forecasting plaque stability depend on the capacity to differentiate between various plaque components. The outermost layer of atherosclerotic plaques, known as the fibrous cap, is essential to the integrity of the plaque. While a weak or ruptured fibrous cap is a major sign of plaque fragility, a thick, unbroken fibrous cap is typically linked to stable plaques. Due to its high collagen concentration and comparatively low water content, the fibrous cap looks hyperintense (bright) on T_1_ imaging. T_2_ scans, on the other hand, can occasionally display a more intricate structure of the fibrous cap, especially in cases of severe inflammation or edema [65]. Another important element of atherosclerotic plaques is the lipid core, which is linked to plaque instability. Due to its relatively low water content, the lipid-rich necrotic core may look hypointense (black) on T_1_ imaging. The lipid core may appear hyperintense (bright) on T_2_ imaging, particularly when there is a high water content or when the plaque contains inflammatory activities. Calcifications, or regions of mineral deposition inside the plaque, are frequently seen in atherosclerotic plaques [66,67]. Because calcified regions have very little water content and do not generate a strong MRI signal, they look hypointense (black) on both T_1_ and T_2_ imaging. Since calcification may indicate an advanced state of atherosclerosis, its detection is crucial. T_1_ and T_2_ MRI are helpful for determining the general size and volume of plaques, as well as for defining their constituent parts [68]. Determining the degree of atherosclerosis and forecasting the likelihood of unfavorable cardiovascular events require this knowledge. The volume of plaque constituents (such as the lipid core, fibrous tissue, and calcifications), as well as the total plaque size, can be measured using both T_1_ and T_2_ sequences. This can assist medical professionals in determining the extent of artery stenosis and forecasting the risk of plaque rupture. T_1_ and T_2_ MRI measurements of the ratio of fibrous tissue to lipid core size have been shown in studies to be a useful indicator for plaque vulnerability [69,70]. MRI can be used to characterize plaque, track the development of atherosclerosis over time, and evaluate the results of therapies meant to lessen plaque burden or increase plaque stability. Changes in plaque volume and composition over time can be monitored using T_1_ and T_2_ MRI. Plaque stabilization may be indicated by an increase in fibrous tissue or a decrease in the lipid-rich necrotic core, whereas plaque destabilization may be suggested by an increase in inflammation or bleeding [71,72]. Additionally, MRI can be used to evaluate the effectiveness of therapeutic therapies, including lifestyle changes, anti-inflammatory medications, and statin therapy. T_1_ and T_2_ imaging can be used to track changes in plaque composition, such as a decrease in lipid core volume or an increase in fibrous tissue, to evaluate the effectiveness of treatment. MRI has limits in terms of resolution and penetration despite its many benefits, including non-invasiveness and superior soft tissue contrast [73,74]. However, T_1_ and T_2_ MRI will remain crucial for the diagnosis, tracking, and treatment of atherosclerosis due to continuous improvements in MRI technology and image processing, which will ultimately enhance cardiovascular risk assessment and patient outcomes [75]. OCT creates detailed images using light waves rather than sound waves, but it works on the same principles as ultrasound. To create photographs, infrared light is emitted into tissues, and the reflected light is measured. OCT offers a far more detailed image than other imaging modalities, like intravascular ultrasonography, because of its strong penetration of infrared light, which enables it to examine arterial wall features with micron-level resolution (10–20 microns) [76]. OCT is frequently used for retinal imaging in ophthalmology, but it has also found important uses in cardiology, especially in the evaluation of coronary artery disease and atherosclerosis. This technology is a vital tool for identifying and describing atherosclerotic plaques because it makes it possible to see the artery wall at the microscopic level [77]. Even at the level of specific components like the fibrous cap, lipid core, and calcifications, OCT’s capacity to generate high-resolution images—typically ranging from 10 to 20 microns—allows for the thorough evaluation of plaque morphology. Plaques with big lipid cores, fragile plaques with thin fibrous crowns, and stable fibrous plaques can all be distinguished using OCT. The risk of rupture and thrombosis varies for these kinds of plaques [78]. One important factor influencing the stability of an atherosclerotic plaque is its fibrous cap. An increased risk of acute events and plaque rupture is linked to a thin or ruptured fibrous cap. OCT is very useful for determining the thickness and integrity of the fibrous cap, which is crucial for estimating the risk of plaque rupture. A thorough examination of the makeup of a plaque is made possible by OCT’s ability to distinguish between distinct elements of the plaque, including the lipid-rich necrotic core, fibrous tissue, and calcifications. This can assist in locating susceptible plaques that have a higher risk of causing unfavorable cardiovascular events [79]. OCT can also be used in the long-term evaluation of atherosclerosis, assisting medical professionals in tracking the disease’s development and the effectiveness of interventional therapies, such as balloon angioplasty or stenting, lifestyle changes, and statins. The early detection of any decline in plaque stability is made possible by the capacity of repeated OCT imaging to monitor changes in plaque size, shape, and composition over time [80]. OCT can be used to validate the expansion and location of the stent, evaluate the existence of any residual plaque or dissection, and evaluate the success of stent insertion following an interventional treatment, such as percutaneous coronary intervention. The propensity of vulnerable or high-risk plaques to rupture, resulting in thrombus formation and acute coronary events like myocardial infarction (MI), is one of their defining characteristics [81]. One of the main objectives of atherosclerosis imaging is to identify susceptible plaques, and OCT has shown itself to be especially effective in this regard. It has been demonstrated that OCT is quite successful in precisely recognizing these traits, which is essential for early intervention to avert disastrous consequences. OCT has drawbacks, including limited tissue penetration and high cost, although it offers substantial benefits in plaque characterization, particularly when evaluating fibrous cap integrity and plaque composition [82]. However, OCT has a lot of potential to help with atherosclerosis diagnosis, management, and treatment, especially when it comes to spotting fragile plaques that are likely to burst. OCT technology is expected to play a bigger part in clinical cardiology as it develops further, providing even more potent instruments for the early identification and treatment of cardiovascular disorders.

### Limitations

The presented results are based on 14 plaques taken from seven patients during surgical endarterectomy due to significant/critical carotid artery stenosis. The number of patients in the study pool may limit the final results, regardless of inclusion criteria, for atherosclerotic plaques in carotid arteries leading to ischemic stroke. Patients with a history of supraventricular arrythmias, such as atrial fibrillations and low ejection fraction due to myocardial infarction, were excluded from the study pool in order to concentrate only on ischemic stroke due to significant/critical carotid artery stenosis. The selection of the contrast agent (Joheksol) Omnipaque was intentional due to the size of the medium particle and its wide availability and use in interventional radiology.

It is critical to acknowledge the study’s limitations, especially regarding sample size, as these could impact how broadly the findings can be applied. Stronger findings might have been obtained with a larger sample. Furthermore, there was little study of related factors, and a more thorough investigation of possible confounders might have produced more complex findings. Lastly, the study only looked at atherosclerosis in carotid arteries; a more thorough understanding of cardiovascular disorders would be possible if a wider range of lesion types were considered. However, we wanted to show that T_1_ and T_2_ relaxation durations might be used to research atherosclerosis. Since MRI imaging does not cause harm to human health, it is a very useful study that may be carried out repeatedly without waiting periods.

## 5. Conclusions

The average of T_1_ and T_2_ relaxation times before and after Joheksol (Omnipaque 350) implementation may be helpful in recognizing the most favorable features of atherosclerotic plaques before scheduled PDT therapy. Further experimental research in animal models may deliver an evaluation of the effectiveness of possible therapy.

In summary, OCT and MRI together provide valuable compositional and morphological information related to changes in articular cartilage at the early stages of osteoarthritic degradation. Together, these techniques provide a powerful complementary approach that results in a comprehensive picture of molecular and biophysical changes in atherosclerotic plaques.

## Figures and Tables

**Table 1:** Study group characteristics.

Disease or Case	n/N (%)
Age, year (mean, range)	76 (69–81)
Male/female gender	5/2
Cardiovascular risk factors	
Hypertension	7/7 (100%)
Diabetes mellitus	5/7 (71.4%)
Dyslipidemia	7/7 (100%)
Smoking history	5/7 (71.4%)
Chronic kidney disease	3/7 (42.8%)
Cardiovascular comorbidities	
Previous myocardial infarction	2/7 (28.6%)
Previous percutaneous coronary intervention	5/7 (71.4%)
Previous coronary artery bypass surgery	1/7 (14.3%)
History of stroke	7/7 (100%)
Normal sinus rythm	7/7 (100%)
Medications	
Statin	7/7 (100%)
Beta-blocker (B-blocker)	6/7 (85.7%)
Aspirin (ASA)	7/7 (100%)
Clopidogrel	2/7 (28.6%)
ACE inhibitors (ACE-i)	5/7 (71.4%)
Angiotensin II receptor blockers (ARB)	1/7 (14.3%)
SGLT2 inhibitors (Flozin)	2/7 (28.6%)

**Table 2:** The average of T_1_ and T_2_ relaxation times on each step of the experiment: before Omnipaque 350 implementation and 1 h and 48 h after Omnipaque 350 implementation. Significant variables *p*-value < 0.05 are highlighted with a star *.

Mean ± SD		
Relaxation Time	T_1_ [ms]	T_2_ [ms]
Before Omnipaque 350 implementation	1231 ± 12 *	63 ± 23 *
1 h after	673 ± 24 *	45 ± 6 *
48 h after	460 ± 45 *	33 ± 4 *

**Table 3:** Values of T_1_ and T_2_ relaxation times of Atherosclerotic Plaque Samples. Significant variables *p*-value < 0.05 are highlighted with a star *.

Atherosclerotic Plaque Samples (n = 14) Before Omnipaque 350 Implementation			Atherosclerotic Plaque Samples (n = 14) 1 h After Omnipaque 350 Implementation		Atherosclerotic Plaque Samples (n = 14) 24 h After Omnipaque 350 Implementation	
No.	T_1_ [ms]	T_2_ [ms]	T_1_ [ms]	T_2_ [ms]	T_1_ [ms]	T_2_ [ms]
1	1163	58	674	45	461	33
2	1271	54	675	47	455	32
3	989	76	679	43	472	28
4	1234	79	585	45	467	31
5	1263	67	684	47	460	29
6	1370	68	673	51	467	35
7	1191	45	677	43	457	28
8	1278	67	679	40	467	35
9	1324	64	698	44	435	38
10	1297	65	701	45	460	45
11	1298	67	785	45	474	27
12	1341	57	654	41	449	33
13	989	56	658	48	469	34
14	1239	64	662	47	464	34
Mean ± SD	1231 ± 12 *	63 ± 23 *	673 ± 24 *	45 ± 6 *	460 ± 45 *	33 ± 4 *

## Data Availability

The original contributions presented in this study are included in the article. Further inquiries can be directed to the corresponding authors.

## Abbreviations

The following abbreviations are used in this manuscript:

CT	Computed tomography
MRI	Magnetic resonance imaging
OCT	Optical coherence tomography
ms	Milliseconds
